# Discovering potential pathways between type 2 diabetes mellitus and diabetic retinopathy: A big data analysis of the South Korean National Sample Cohort

**DOI:** 10.1097/MD.0000000000034576

**Published:** 2023-08-04

**Authors:** Yoojoong Kim, Changwan Hyun, Minhyeok Lee

**Affiliations:** a School of Computer Science and Information Engineering, The Catholic University of Korea, Bucheon, Korea; b Department of Urology, Korea University College of Medicine, Seoul, Korea; c School of Electrical and Electronics Engineering, Chung-Ang University, Seoul, Korea.

**Keywords:** big data, diabetes mellitus, diabetic retinopathy, disease network, retinal disorders

## Abstract

Diabetes mellitus, a prevalent metabolic disorder, is associated with a multitude of complications that necessitate vigilant management post-diagnosis. A notable complication, diabetic retinopathy, could lead to intense ocular injury, including vision impairment and blindness, due to the impact of the disease. Studying the transition from diabetes to diabetic retinopathy is paramount for grasping and halting the progression of complications. In this study, we examine the statistical correlation between type 2 diabetes mellitus and retinal disorders classified elsewhere, ultimately proposing a comprehensive disease network. The National Sample Cohort of South Korea, containing approximately 1 million samples and primary diagnoses based on the International Statistical Classification of Diseases and Related Health Problems 10th Revision classification, was utilized for this retrospective analysis. The diagnoses of both conditions displayed a statistically significant correlation with a chi-square test value of *P* < .001, and the *t* test for the initial diagnosis date also yielded a *P* < .001 value. The devised network, comprising 27 diseases and 142 connections, was established through statistical evaluations. This network offers insight into potential pathways leading to diabetic retinopathy and intermediary diseases, encouraging medical researchers to further examine various risk factors associated with these connections.

## 1. Introduction

Diabetic retinopathy is a frequent complication of diabetes, resulting in harm to the retina and nerves. It is more prevalent in type 1 diabetes patients than those with type 2 diabetes and is present in a majority of individuals with prolonged diabetes. Thus, diabetic retinopathy is a key cause of blindness in developed nations with high diabetes incidence. For example, it was observed that in the United States, there were 5.5 million individuals diagnosed with diabetic retinopathy in 2005, a number projected to reach 16.0 million by 2050.^[1]^ The escalating diabetes prevalence is likely to result in a proportionate increase in the incidence of diabetic retinopathy.

The genesis and risk factors of diabetic retinopathy have been extensively researched, with a focus on biochemical and environmental origins. Persistent high blood sugar levels result in the formation of advanced glycation end products and the stimulation of protein kinase C, both of which contribute to damage to the retina’s blood vessels.^[2,3]^ Hypertension causes harm to the retinal vessels by amplifying the shear stress on the vessel walls, resulting in endothelial dysfunction, heightened permeability, and blood vessel blockage.^[4,5]^

Besides hyperglycemia and hypertension, multiple other risk factors have been identified for diabetic retinopathy. For type 1 diabetes patients, puberty and pregnancy are recognized as risk factors for the onset and progression of diabetic retinopathy.^[6]^ Smoking has also been highlighted as a risk factor for diabetic retinopathy, with evidence indicating that smokers have an elevated risk of developing the condition and experiencing more severe forms of the disease.^[7]^ Obesity and dyslipidemia are also correlated with an increased risk of diabetic retinopathy, likely due to their role in insulin resistance and the subsequent metabolic irregularities that can harm the retina.^[8]^

Contemporary research has also indicated a potential connection between sleep apnea and diabetic retinopathy.^[9,10]^ Sleep apnea, a disorder marked by breathing disruptions during sleep, can cause intermittent hypoxia and oxidative stress, both implicated in the onset of diabetic retinopathy. Studies reveal that patients with sleep apnea possess a higher risk of developing diabetic retinopathy, and that the management of sleep apnea with continuous positive airway pressure could mitigate the risk of the condition’s development.^[11]^

Beyond environmental risk factors, genetic elements also contribute to the emergence of diabetic retinopathy. Variations in several genes, including those involved in the renin-angiotensin-aldosterone system, the complement system, and oxidative stress pathways, have been correlated with a heightened risk of the disease.^[12,13]^

In this paper, we propose a causal network for diabetic retinopathy, utilizing a retrospective perspective and big data analysis. We pinpointed statistically significant historic disease factors linked with diabetic retinopathy using a vast patient dataset with a history of the condition. This methodology facilitated the exploration of the relationships between different disease factors and the identification of possible causal pathways underlying the disease’s development and progression. Through a deeper understanding of the risk factors and underlying mechanisms of diabetic retinopathy, we aim to establish more effective strategies for prevention, early detection, and treatment of this debilitating disease.

## 2. Materials and methods

### 2.1. Dataset

This was a retrospective study using cohort data. The National Health Insurance Sharing Service in South Korea has provided the National Sample Cohort that contains various clinical factors of 1 million people extracted by random sampling from 2002 to 2015 for the purpose of research.^[14]^ The dataset consists of about 2 billion medical events such as the date of diagnosis with disease codes, the prescription history, and health screenings. Ethical review was waived for this study with the approval of the institutional review board of Chung-Ang University (IRB no. 1041078-202104-HRBM-101-01) because of the use of a retrospective study design. All analyses were performed with R.

### 2.2. Processing the National Sample Cohort dataset

**Figure 1.**
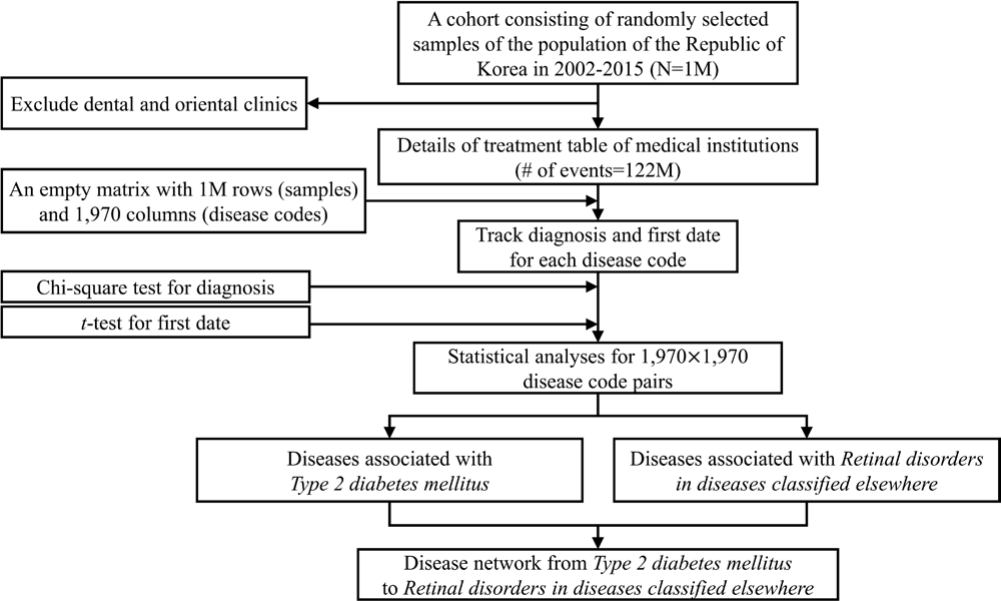
Flow diagram of the study.

Figure 1 presents the flow of the study. Among the whole data, the *details of treatment* table of medical institutions excluding dental and oriental clinics was used in this study. The *details of treatment* table contained 122M events with the encrypted person ID, the disease code of the main diagnosis, and the date of the corresponding insurance history. The disease code followed the Korean Standard Classification of Diseases 8 translated from the International Statistical Classification of Diseases and Related Health Problems 10th Revision code. We simplified the disease codes of the main diagnosis in the 3-character code consisting of 1 letter and 2 numbers according to International Statistical Classification of Diseases and Related Health Problems 10th Revision.

### 2.3. Historical diagnosis dataset

We considered the first insurance records for each disease code as an event of interest. Because a patient can be diagnosed with a single disease code multiple times, the first time of diagnosis was considered for the analysis. We derived the time point when patients were first diagnosed with each disease code by retrieving the medical history for each patient. We tracked all dates each sample was diagnosed by disease code and collected the earliest date for each disease. We constructed a dataset composed of 1M samples and 1970 disease codes.

### 2.4. Statistical analyses

Two types of statistical tests were performed on all possible pairs of 1970 disease codes. We considered the statistical significance of the gap time between the 2 disease codes through the *t* test for a risk factor. In addition, the chi-square test was performed to analyze the association between the 2 disease codes. We selected pairs of disease codes that showed statistical significance (*P* < .01) for both statistical tests.

### 2.5. Constructing disease network

Through the analyses, disease codes statistically associated with each of the 2 diseases were selected. Among them, disease codes that showed statistical significance for both diseases were first considered for composing a network. In addition, disease codes that can configure pathways with indirect associations were also considered. Finally, we constructed a disease network from *Type 2 diabetes mellitus* to *Retinal disorders in diseases classified elsewhere* among pairs of disease codes.

## 3. Results

We propose a disease network between *Type 2 diabetes mellitus* and *Retinal disorders in diseases classified elsewhere*. The proposed disease network was constructed based on a statistical analysis using the national cohort data with 1M samples and 1970 disease codes.

### 3.1. Characteristics

Table 1 shows the characteristics of study samples for 2 disease codes of interest. Among the total samples of the cohort data, 74,328 were diagnosed with *Type 2 diabetes mellitus* and 14,575 were diagnosed with *Retinal disorders in diseases classified elsewhere*, respectively. Among the samples diagnosed with *Type 2 diabetes mellitus*, 51.8% were male, and sex showed statistical significance in the diagnosis. Among samples diagnosed with *Retinal disorders in diseases classified elsewhere*, the proportion of males was 49.8%, similar to that of undiagnosed samples. The proportion of those over 60 years of age among the diagnosed samples was higher than the proportion of undiagnosed samples for both disease codes.

**Table 1:** Characteristics of samples for *Type 2 diabetes mellitus* and *Retinal disorders in diseases classified elsewhere*.

Disease code	Diagnosed		Undiagnosed		*P* value
	N	%	N	%	
Type 2 diabetes mellitus	74,328	–	1,005,277	–	–
Sex					
Male	38,493	51.8	501,347	49.9	<.001
Female	35,835	48.2	503,930	50.1	
Age					
0–59	22,985	30.9	804,191	80.0	<.001
60-	51,343	69.1	201,086	20.0	
Retinal disorders in diseases classified elsewhere	14,575	–	1,065,030	–	–
Sex					
Male	7256	49.8	532,584	50.0	.599
Female	7319	50.2	532,446	50.0	
Age					
0–59	3817	26.2	823,359	77.3	<.001
60-	10,758	73.8	241,671	22.7	

### 3.2. Statistical association

Table 2 presents the number of diagnoses for the disease code pair and the statistical significance based on the chi-square test. The number of people diagnosed with both diseases was 11,842. The chi-square test for the contingency table showed a statistical significance of *P* < .001. In addition, the diagnosis time of *Type 2 diabetes mellitus* precedes *Retinal disorders in diseases classified elsewhere* as 812 days on average. The *t* test for the diagnosis time showed *P* < .001 as well. The result showed a statistical association between *Type 2 diabetes mellitus* and *Retinal disorders in diseases classified elsewhere*.

**Table 2:** Contingency table and statistical significance for *Type 2 diabetes mellitus* and *Retinal disorders in diseases classified elsewhere*.

		Retinal disorders in diseases classified elsewhere		*P* value
		Diagnosed	Undiagnosed	
Type 2 diabetes mellitus	Diagnosed	11,842	62,486	<.001
	Undiagnosed	2733	1,002,544	

### 3.3. Disease network

**Figure 2.**
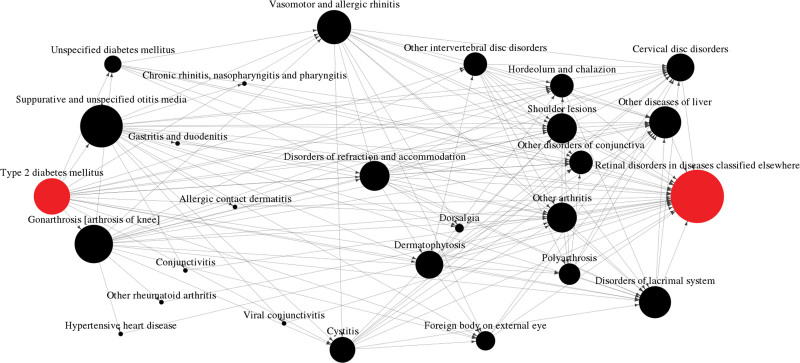
The disease network from *Type 2 diabetes mellitus* to *Retinal disorders in diseases classified elsewhere.*

Figure 2 shows the proposed disease network. The proposed disease network was composed of 27 nodes and 142 edges. In the network, a node represents a disease code and an edge represents a linkage between 2 disease codes based on statistical analysis. *Type 2 diabetes mellitus* showed 17 linkages as the starting point of the pathway in the network. *Retinal disorders in diseases classified elsewhere* showed 25 linkages as the final point of the network. The size of nodes reflects the number of adjacent nodes with edges. Otitis media showed the highest number of adjacent nodes with 20 besides the 2 disease codes. Seven disease codes in the network had 2 adjacent nodes as the minimum.

## 4. Discussion and conclusions

The proposed networks imply that otitis media could be a potential intermediary between type 2 diabetes and retinal diseases. While cervical disc irregularities may not be directly linked with diabetic retinopathy, individuals with such conditions may be more susceptible to additional comorbidities that increase their vulnerability to diabetic retinopathy.

Hyperglycemia and obesity are among the most significant risk factors for type 2 diabetes, both being connected with immunological dysfunction and susceptibility to infections.^[15]^ Individuals with type 2 diabetes possess a higher risk of infections,^[16,17]^ including otitis media, due to likely immune system degradation caused by elevated blood sugar levels. Patients with diabetes mellitus often experience a higher frequency and severity of infectious diseases, which can boost morbidity and mortality rates. The increased prevalence of infections in diabetic patients can be attributed to a hyperglycemic environment that fosters immune dysfunction, such as compromised neutrophil function, antioxidant system depression, and humoral immunity. Additionally, micro and macro-angiopathies, neuropathy, reduced antibacterial activity in body fluids, and a greater number of medical interventions can all contribute to the higher infection risk in diabetic patients. Infections can affect all organs and systems, but some, including malignant external otitis, and gangrenous cholecystitis, are more commonly observed in diabetic individuals. These infections not only lead to increased morbidity but may also signal the initial presentation of diabetes mellitus or a contributing factor to the disease’s inherent complications.^[17]^ Therefore, individuals with diabetes may be more prone to contracting otitis media. Furthermore, obesity is a risk factor for both type 2 diabetes and otitis media, and overweight or obese children are more susceptible to ear infections than their non-obese counterparts.^[18]^ Although current evidence regarding the association between type 2 diabetes and otitis media is limited, additional research is necessary to fully explore potential links.

Nonalcoholic fatty liver disease (NAFLD), marked by fat accumulation within the liver, has been correlated with an increased risk of diabetic retinopathy.^[19–22]^ This association may be due to shared metabolic complications common to both disorders, such as obesity, insulin resistance, and dyslipidemia. Moreover, cirrhosis and hepatitis C virus (HCV) infection have been linked with a heightened risk of diabetes,^[23–26]^ which is a significant risk factor for diabetic retinopathy. The results of a study involving 9841 individuals with type 2 diabetes showed that 8.4% had the condition and 2.1% were positive for anti-HCV.^[25]^ The probability of developing type 2 diabetes was elevated among those who were older, nonwhite, had a high body mass index, and low socioeconomic status, whereas it was lower among those who reported prior illicit drug use. Individuals with liver conditions such as NAFLD, cirrhosis, or HCV infection may face an increased risk of diabetic retinopathy due to shared metabolic issues or the presence of diabetes. Moreover, specific medications for liver disease could have detrimental effects on the eye and heighten the risk of diabetic retinopathy. For instance, interferon treatment for HCV infection has been connected to the onset of diabetic retinopathy.^[27,28]^

Although cervical disc disorders and diabetic retinopathy may not be directly linked, they could be related within the proposed network. Indeed, individuals with cervical disc anomalies might face a higher risk of developing diabetic retinopathy due to mutual risk factors. Factors such as obesity, hypertension, and diabetes are acknowledged risk contributors for diabetic retinopathy and are often associated with cervical disc diseases. Some drugs used to manage cervical disc disorders, like corticosteroids, may also escalate the incidence of diabetic retinopathy.^[29–31]^ While further research is needed, this potential link may assist in identifying individuals at higher risk of developing diabetic retinopathy.

For patients with type 2 diabetes, the link between arthritis and diabetic retinopathy remains underexplored. Nonetheless, studies suggest a possible connection between arthritis and diabetes, specifically that individuals with rheumatoid arthritis, an inflammatory form of arthritis, may have a higher likelihood of developing type 2 diabetes.^[32,33]^ Hyperglycemia, hypertension, dyslipidemia, and kidney disease are the most common risk factors for diabetic retinopathy, frequently present in type 2 diabetes patients. Therefore, those with both arthritis and type 2 diabetes may have a heightened risk of developing diabetic retinopathy due to these shared factors. While no direct connection between arthritis and diabetic retinopathy in type 2 diabetes patients is firmly established, further investigations are needed to uncover the potential mechanisms linking the 2 conditions.

Lacrimal system disorders, such as dry eye syndrome, are common among diabetic individuals, particularly those with unregulated blood sugar levels. Dry eye syndrome can lead to an array of symptoms, like discomfort, blurred vision, and eye infections. Furthermore, recent investigations suggest that individuals with dry eye syndrome may have an increased risk of developing diabetic retinopathy. For instance, a study involving 199 participants found that 108 patients (54.3%) had dry eye syndrome.^[34]^ While dry eye syndrome was more prevalent in older and female patients, this correlation was not statistically significant. However, there was a significant association between the duration of diabetes and dry eye syndrome, and the syndrome was more prevalent in diabetic patients with diabetic retinopathy. While the exact mechanism underpinning this relationship is not fully comprehended, inflammation and oxidative stress, both implicated in dry eye syndrome and diabetic retinopathy, might play a role. Additional studies are required to elucidate this connection and identify potential interventions to prevent or manage both conditions.

This investigation points to possible connections between several medical conditions and the onset of diabetic retinopathy. Conditions such as otitis media, cervical disc disorders, NAFLD, liver cirrhosis, HCV infection, arthritis, and lacrimal system disorders are all hypothesized to correlate with diabetic retinopathy. Shared risk factors like obesity, hypertension, hyperglycemia, and dyslipidemia could play a part in these associations. While further research is needed to confirm these links, recognizing individuals at higher risk of developing diabetic retinopathy could facilitate early detection and intervention, thus helping to prevent visual impairment.

## Author contributions

**Conceptualization:** Minhyeok Lee.

**Formal analysis:** Yoojoong Kim.

**Funding acquisition:** Yoojoong Kim.

**Investigation:** Yoojoong Kim, Changwan Hyun.

**Methodology:** Yoojoong Kim, Changwan Hyun, Minhyeok Lee.

**Resources:** Minhyeok Lee.

**Supervision:** Minhyeok Lee.

**Validation:** Yoojoong Kim.

**Visualization:** Yoojoong Kim.

**Writing – original draft:** Yoojoong Kim, Minhyeok Lee.

**Writing – review & editing:** Changwan Hyun, Minhyeok Lee.
